# To the Friends of Cincinnati Medical Reform in Ohio and the West Generally

**Published:** 1835

**Authors:** Daniel Drake

**Affiliations:** Cincinnati


### To the Friends of Cincinnati Medical Reform, in Ohio and the West generally.

The object for which you have labored, an improved condition of the Medical College of Ohio, has as you have just read, not been attained, and a new school has been organized. No one pretends to doubt, that the legislature of Ohio, in appointing a new Board of Trustees, had distinctly in view, a reformation of the College. The professors, however, did not think proper to resign, and some of those whom it was generally expected would not be thought qualified for retention, have been kept in, because eight votes out of eleven could not be got to replace them with abler men. It is far from being my intention to question the judgment of the gentlemen, all highly respectable and intelligent, who thought four out of six of the Faculty worthy of being retained; but I am as little disposed to admit that, I myself, am censurable; for not thinking in the same way, or for accepting a professorship in another corporation. If the Medical College of Ohio had been prosperous, such a project would not have been undertaken, at least by those who *have* engaged in it. As it is not prosperous, no harm can come to the interests of the profession from a new school. The Cincinnati College has taken nothing from the Medical College of Ohio, and, therefore, deprives our students of medicine of no advantages which that institution could afford. As to anatomical subjects, it is well known that enough are attainable for two schools. Then, who is or can be injured by the new school, but the professors of the old? the fear of which, perhaps, may impel them to greater efforts than heretofore, and thus the students who choose to return to that college will be better taught—for which they may thank the new project.

It is unquestionably not the policy of the state to grant a monopoly of any kind, and especially one, which is charged with the interests of education. We can, at this moment, recollect no less than six chartered institutions which have the *franchises* necessary to the creation of Medical Faculties, and there may be several others. A number of these have been chartered since the establishment of the Medical College of Ohio. It is the policy of the state, then, to encourage salutary emulation, to multiply the places and means of instruction, and to raise our young men above a dependence on a single institution, which becoming defective, may disappoint their hopes. Indeed, the constitution of Ohio expressly requires of the legislature to set its face against all monopolies in education, and instructs them to afford equal chartered privileges to all the citizens of the state, in the following section of Art. 8th:

"Sec. 27. That every association of persons when regularly formed within this state, and having given themselves a name, may, on application to the legislature, be *entitled* to receive letters of incorporation, to enable them to hold estates, real and personal, for the support of their schools, academies, colleges, universities, and other purposes."

Such has been the policy of the state for a third of a century—that is, for an age; such is the supreme law of the land; such the understanding which all our legislatures have had of their duty;—and if so, may not corporations already in existence, and even private associations of individuals, regulate themselves by the same principles? I will illustrate this matter by a few examples.

Within two miles of this city, there is an incorporated institution entitled Lane Seminary, expressly designed for Presbyterian theological studies. Now what would have been thought, if its professors and trustees had opposed the recent establishment of another school of the same kind, by the trustees of Oberlin College, on Lake Erie? or what would be thought of Dr. Beecher and his colleagues, if they were to set out and oppose the establishment of a theological department in the Cincinnati College, to be placed under the direction of Dr. Wilson? Again, suppose that a law school had existed in Cincinnati, the teachers of which were so incompetent, that the Bar of Ohio sent their pupils to the schools of Lexington and Litchfield, would society have frowned upon Judge Wright, Mr. King, and Mr. Walker, for setting up a better one, and drawing law students into Ohio, while the other drove them out? Still further, our legislature, several years ago, chartered an Insurance company in this city, and since have chartered two others; but who ever heard that Mr. Neville and his associates in the first, were disposed or felt themselves at liberty, to remonstrate against the establishment of the others? Finally, after the Ohio Mechanics' Institute was chartered by the legislature, that honorable body, on application, created the Cincinnati Lyceum, both designed for popular instruction, by means of public lectures on any branch of human knowledge; but did Mr. Foote, Mr. Graham, Mr. Fletcher, and Mr. Bonsall, oppose this proceeding? or will any one pretend they ought to have interfered? I think not, and still, as the Institute was prosperous, they would have had more right and reason on their side, than would belong to the Trustees of the Medical College of Ohio, should they oppose the establishment of a second medical school, when they have failed to give respectability and usefulness to the first.

The professors of our *alma mater*, the University of Pennsylvania, were violently opposed to the institution of the Jefferson Medical College; but the legislature of Pennsylvania, governed by broader views, went ahead; and what has been the effect, but the assemblage, last winter, in Philadelphia, of 600 pupils, when something more than 400 had been the most that had ever attended before the organization of the second school! And who will say, that the lectures in the University, have not been made more spirited and instructive by the rivalry?

One third of the population of the United States is in the Valleys of the Mississippi and the Lakes, while the whole region has but two schools, one of which has been pronounced by the profession of the state whose name it bears, and by a large majority of the physicians of the city in which it has been in operation for fifteen years, and by its own Trustees, to be utterly defective—a sentence which is confirmed by the small number of its pupils. Is there no need, then, of reform, or of a better school? Shall there be fourteen medical schools among eight millions of people in the other states, and a ban put upon those who would have more than two, for four millions in the West, when one of these two is so unattractive as to be the ninth or tenth school in the Union, in the number of its pupils? Kentucky, with but two-thirds of the population of Ohio, gave to her own school, last winter, one hundred and twenty students, at least double the number of *all* the pay pupils in the Ohio school from every quarter! I state as a fact, that a medical gentleman declared, at the Columbus convention of physicians, in January, that he knew, in the county in which he resided, and in an adjoining county, no less than eight students of medicine, who were in attendance on lectures in eastern schools! And this too after our state had given thirty-six or thirty-seven thousand dollars for the establishment of a college at home. Shall it be said that this college has not yet attained to puberty? It may not in functions, but certainly has in years. Early and rapid development is the constant effect of the warm moral and social climate of the West; and where there is any thing to come forth we see it unfolded betimes. Thus the third class of the Kentucky school comprised 138 pupils, which is more than the Ohio school has yet numbered. The sixth class of the former reached 282—the twelfth of the latter was 200 less! When the 282 assembled, they found themselves in what had been the bar room of a tavern!—there was no public edifice—the state had not, and has not since, given but $5000; while all the circulars of our college have set forth the munificence of Ohio, and the magnitude and splendor of the college edifice, the attractive front of which, has more than once been engraved, at the expense of the state, and sent abroad in books, and newspapers, as a substitute for the fame of the professors. Unfortunately for those who relied on this artifice, students attend medical schools to be instructed, not to sit in splendid edifices;—to listen to the eloquence of professors—not to admire the beauty of the columns, which adorn the empty and echoing halls. The Jefferson Medical College was instituted in 1825, five years after the Medical College of Ohio; without any endowment from the state of Pennsylvania; and under the very walls of the venerable and popular university, which is honored with the name of the state, and cherished by the wealth and moral power of the city; and still, with all these disadvantages, it has already attained to three times the number of the Ohio college, placed in a spot more favorable to the building up of a great school, than almost any other in the Union.

It has been said, however, that I ought to have re-entered the college, and waited for its expurgation at a future time. But if it had not flourished after I went in, would not my friends have been taunted with the failure, and mortified at the result? If it had flourished even to a moderate extent, would the Board of Trustees *then* have thought of making further removals? And could any removals have been attempted, without involving me in the charge of having instigated them? The fact was and is, that if I had entered the school with a particular set of colleagues, it would have been my duty to sustain them. While out of the institution, I might co-operate with the friends of reform, but once in, I must stand up for my associates. On all these points, I have not been allowed to judge for myself. I have been erected into a phenomenon—a teacher able to raise the school into immediate distinction, but still unqualified by my talents and sagacity to judge whether its organization was such as would be successful! I have been made a very Hercules—capable of performing wonders, but incapable of judging when or where, they should be wrought. Not claiming, however, to wield the club of Hercules, I do not entirely adopt his character for stupidity. Indeed, I consider myself better qualified to judge of the means necessary to success in a medical school, than to teach one of the departments; and think that those respectable gentlemen, not of the medical profession, who have done me the honor to say, that I would be a great acquisition to the College, might, without inconsistency, have admitted, that such an one must have some ability to judge whether its organization was good. They have, however, thought otherwise, and a new school is the first fruits of their decision.

So much for the necessity of a revolution in the affairs of one college, and in explanation of the reasons for instituting another school. But it is said, here in the city, by those interested in keeping themselves in the college, that the new project is ephemeral and designed merely for effect. I reply, that it *is* ephemeral, but in the same sense that a nation is ephemeral, compared with the human race; and that it is, in truth, for effect—the effect of assembling together those pupils who desire to study the science profoundly, and of teaching them with diligence. It is bruited abroad still further, that the undertaking is another manifestation of "*Drake's unhallowed ambition;*" to which I reply, that it would be well for the profession, if the charge of ambition could be justly brought against some of his cotemporaries. Yet farther, it has been insinuated, that I have deluded a few persons into the measure, and that they will soon get their eyes open to the dangers of the enterprise, and abandon it. In answer, I state that I came into the project on the persuasion of many respectable persons, both in and out of the profession—of the country as well as the city—and that they are quite in earnest. All who doubt this assertion, need but await the rapid development of events to be convinced of its truth. Some unforseen obstacles may *perhaps* lie in the way of this development, and require to be thrust aside, or trampled under foot. Well! be it so. I have lived too long in the West to need a macadamized road for any journey, and look upon the difficulties that may be encountered as mere stimulants to effort, like the wounds inflicted by the thorns and nettles of the thicket, which quicken the footsteps of the young Backwoodsman, but never "turn him on his heel."

The new school, it is said, will put the physicians of the city by the ears. But why should it? Have our medical gentleman no more dignity and self respect, than to allow such an event to involve them with each other? For what purpose should they go into a quarrel? If there must be quarrelling, let those who are to enjoy the spoils of victory carry on the foray. Let the professional and other gentlemen of the city, harmoniously mount the scaffold of observation, and amuse themselves with the *Avaritiæ adversus gloriam certamen*, and the quiet of the community will be disturbed in no other way, than by the thunderings of rival lecture rooms: In them the battle must ultimately be decided, and there it might as well begin.

I have been told that I ought to be associated with Dr. Eberle and Dr. Cobb. In turn, I say, Dr. Eberle and Dr. Cobb ought to be associated with me. I am the oldest man, (whatever may be their superior reputation,) came into the West before either of them was born, and founded the College before Dr. Cobb even commenced the study of medicine. Notwithstanding this, for several months past, I sought a union with them, and as they professed a desire to see me in the Medical College of Ohio, repeatedly sent messages, proposing to them to resign and unite with the friends of reform and myself, in building up, in that corporation, a new Faculty, in which they should have the same chairs they now fill; but they were both afraid to relinquish their places, although they declared, they would resign, if at least half their colleagues were not put out. If they wanted moral courage to do what they themselves thought necessary to the consolidation of parties, the establishment of one respectable institution, and the restoration of harmony in the profession, I am not to blame. When the new Faculty of the Cincinnati College was instituted, they were on my own recommendation offered places, which they declined, although they must have known that their acceptance would have been followed by an immediate vacation of the remaining chairs, and a transfer of the newly organized Faculty to the institution they had just left. Thus, I could not become associated with them, except under circumstances which they had declared, would prevent their remaining in that institution! I make this statement that the friends and pupils of those gentlemen may know, that efforts were made to embrace them in a new organization, rather than to deplore their want of concurrence. Neither of them, nor any other single man, is a *sine qua non* to the success of *any* medical institution, and the Cincinnati school *shall* succeed without them. If they should do hereafter, what they have not done heretofore, raise the Medical College of Ohio into respectability, we shall have *two good*, instead of *one bad* school, and the revolution will not have been in vain. There is however, but little prospect of this result, for such is the condition of the Medical College of Ohio, that Dr. Eberle speaks publicly of its depressed and disorganized state, and says, that if its condition should not be much improved he will resign at the end of the next session!

Extended as this address is likely to prove, I cannot refrain from a reference to the origin and history of the Cincinnati College, of which most of the *present* inhabitants of Cincinnati know nothing.

It is impossible, that the surviving founders of this institution, should not rejoice to hear its deserted apartments again vocal with the accents of instruction. The outer walls, with much of the interior work of the vast edifice, were erected by a very general contribution of the citizens of Cincinnati, 20 years ago, when it was designed for popular education. Three or four years afterwards the late Gen. William Lytle, who came into the West, when Ohio was not, and had aided in pursuit of the Indian over the very ground where the edifice now stands, proposed to some of our citizens, in that spirit of munificence which was peculiarly his own, that they should finish the building, endow it and obtain a college charter. Leading the way with a subscription of eleven thousand five hundred dollars, he was followed, by as many respectable citizens as made forty in the aggregate, and their contributions amounted to more than as many thousand dollars. A charter was obtained which gave ample power to appoint professors, organize a Faculty and confer " All the degrees which are usually conferred in any College or university in the United States."

Regular College classes were organized, and many of the prominent young men of Cincinnati, were taught and graduated in the institution. But the full organization of Miami University, ten years ago, with a rich landed endowment by the general government, drew off the youth of this region, and at length the Cincinnati College was suspended. Of the 40 citizens who founded the institution, sixteen are now numbered with the dead, and the families of many of them have fallen into poverty, without even the consolation of seeing, for several years past the slightest public good resulting from the investment of that, which having been withheld would have been most precious to them in these latter days. To the children of those public spirited gentlemen, and to all other contributors from the beginning of the project in 1814, who are now alive, or are represented in their sons and daughters, it cannot fail to be interesting, that what they gave of their hard and honest earnings, will at last be made available to the interests of education.

But there are persons in the community, who would deny them the gratification of seeing the public benefited by their expenditures — by the days and nights of toil they wasted on that object? Shall it be said to them, you must not exercise the powers which the Legislature gave you as a reward for your liberality, because such exercise may lessen the number of pupils in a sinking institution, that dreads nothing so much as honest competition? Has it come to this, that the remnant of the respectable and munificient population of Cincinnati, who, when it was but a village built the first College edifice ever erected in the state, cannot be permitted to make it available to the good of the community, lest it should diminish the income of four incumbents in the Medical College of Ohio, three of whom were not then in the West, while a fourth was living in an obscure corner of Europe, and is not yet it is said naturalized in the United States?

Is this the way that liberty works? Is this the result of that freedom, which creates, and in turn must be sustained by unrestrained emulation? If so, farewell to the noblest schemes which philanthrophy can invent! The genius of civilization has repealed the laws by which he raised up man from savage helplessness, to intellectual and moral strength! He says to the strong, you shall not help yourselves—to the weak, ye are my favorite children! I will defend you from rivalship, and smite your competitors with the rod of my power! I have hitherto permitted the swift to run, and my affections were for those whose march was onward; but, hereafter, I will confer honors upon the unaspiring; I will shower rewards upon the indolent, and my pride shall be in those who loiter by the way!

In connection with the arrangement for a medical department I may mention, for information at home, that the present and the Board of Trustees of last year, have assigned apartments in the edifice to the Cincinnati Medical society, the Cincinnati Literary Society, the Western Academy of Natural Sciences, and the Western College of professional teachers; thus the whole building will henceforth be brought into requisition, and being, as it will put into perfect repair, promises soon to become one of the most beautiful and magnificent edifices of the city—a monument of public spirit, a superstructure consecrated to the public good.

This communication, addressed to the profession in the West generally as well as at home, is more especially designed for the eye of those pupils and medical friends, who have so often, personally and by letter, expressed themselves, in language which I shall not repeat, on the subject of my past relations with the Medical College of Ohio. In the dark days of adversity, which shrouded those relations, their letters came like the occasional sun beams which penetrate the gloom, and fall pleasantly on the eye of him who gropes beneath, although they may not illuminate the path by which he must travel out. The impression they made is imperishable, and will constitute for years to come, a most powerful stimulus to exertion in the halls of medical education, where, with many of those to whom this little effusion is addressed, not a few of the happiest hours of my life have been spent.

In this and a preceding article, I have said much of myself, but let it be remembered, that others have said of me still more. I have often been compelled to grapple, single-handed, with combinations, formidable by their numbers, their influence, and their individual respectability; but, in every instance, up to the commencement of the narrative of which this is a part, I have written in my own defence against published attacks. If in any stage of this protracted controversy, injustice has been done to a single individual, I stand ready to make public reparation; if I have, knowingly, falsified any public document, or perverted any important fact, let me be convicted. Finally, I expect hereafter to write more *on* the profession, and less *of* it; and, instead of uttering my own *complaints*, to speak *on* the *complaints* of others; not, it is true, in the wisdom of an oracle, but with the heart of a man devoted to the glory of his profession.

DANIEL DRAKE, M. D.

Cincinnati, June 30th, 1835.

